# Cytokine Receptor-like Factor 3 (CRLF3) and Its Emerging Roles in Neurobiology, Hematopoiesis and Related Human Diseases

**DOI:** 10.3390/ijms26083498

**Published:** 2025-04-08

**Authors:** Clifford Liongue, Alister C. Ward

**Affiliations:** 1 School of Medicine, Deakin University, Geelong, VIC 3216, Australia; c.liongue@deakin.edu.au; 2 Institute for Mental and Physical Health and Clinical Translation, Deakin University, Geelong, VIC 3216, Australia

**Keywords:** CRLF3, cytokine receptor, immunology, neuroscience, oncology

## Abstract

Cytokine receptor-like factor 3 (CRLF3) has an extended evolutionary history, which has been conserved across metazoan species. It consists of several structural elements, notably including a fibronectin type 3 (FBNIII) domain containing a WSXWS motif that is synonymous with so-called class I cytokine receptors present throughout bilaterial species, and a proposed spl1 and ryanodine receptor (SPRY) domain that represents a widespread protein–protein interaction module. The function of CRLF3 has remained enigmatic, but several recent investigations have revealed critical insights into its biological roles. These studies suggest that CRLF3 principally functions in neural and hematopoietic cells, where it plays critical and diverse roles in the development and function of specific cell populations. Disruption of CRLF3 has also been associated with several human diseases, mainly associated with these same lineages but also including malignancy. The mechanisms by which CRLF3 exerts these diverse effects remain uncertain, although a number of potential options have emerged.

## 1. Introduction

Cytokine receptor-like factor 3 (CRLF3) has a very long evolutionary history. It is present in organisms across the breadth of eumetazoan species, including cnidarians, protostomians and bilaterians, the latter including invertebrates, chordates and higher vertebrates [1,2]. However, a range of species lack CRLF3, such as many insects [3], indicating it is not essential. Expression and functional studies have increasingly indicated roles for CRLF3 in neuronal and hematopoietic cells [4,5,6,7], with its perturbation linked to neurological disorders, immune disruption and various neoplasms [5,8,9]. How CRLF3 exerts these functions remains ambiguous, since it possesses structural and functional characteristics consistent with a classical cytokine signaling function, but also other features that point to alternative intracellular roles. This review aims to summarize the current knowledge base regarding CRLF3 in both normal biology and disease, including an articulation of the potential modes of action underpinning its key functions, as well as pointing to future directions for research.

## 2. Biological Roles of CRLF3

**Figure 1:**
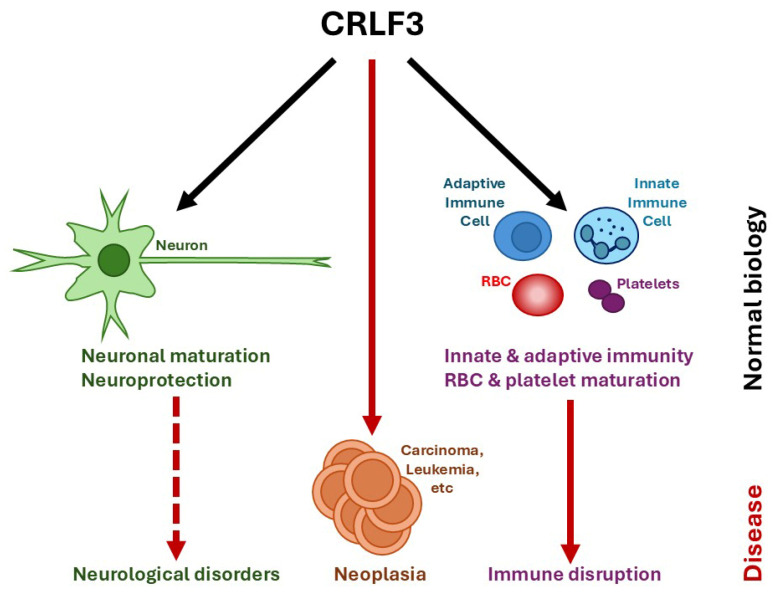
Roles of CRLF3 in health and disease. Schematic view of the various potential functions identified for CRLF3 in normal biology (black solid arrows) as well as in disease states in which CRLF3 is disrupted (red dotted arrows) or overexpressed (thick red solid arrows), with relevant cell types indicated.

Over recent years, several groups studying CRLF3 in a range of biological systems have identified a number of diverse roles for CRLF3 (Table 1). The majority of the phenotypes attributed to CRLF3 relate to neuronal and hematopoietic/immune cells, including relevant diseases (Figure 1).

### 2.1. Neuronal Cells

*CRLF3* gene homologues show expression in various neuronal and sensory tissues across many species. For example, embryonic expression of the zebrafish (*Danio rerio*) *crlf3* homologue was identified in regions of the developing eye and brain [7], while the mouse (*Mus musculus*) *Crlf3* was expressed in the embryonic brain and nervous system [18]. In adults, expression in the brain was identified in the migratory locust (*Locusta migratora*) *crlf3* [3], the brown croaker (*Miichthys miiuy*) *crlf3* [17] and in the central nervous system of the African clawed frog (*Xenopus laevis*) *crlf3* [19], while mouse *Crlf3* was found to be expressed in the mid-brain and sensory organs [18] and human (*Homo sapiens*) *CRLF3* in cerebellum and particularly microglial cells, neurons and dendrites [20].

Consistent with this, functional studies have implicated CRLF3 in specific aspects of neuronal cell development and function. For example, in a rat neuronal cell line model, ablation of CRLF3 resulted in a reduced number of synaptic-like microvesicles (SLMVs), indicative of decreased maturation [10]. In a human iPSC-cerebral organoid model, those derived from individuals possessing a predicted deleterious CRLF3^L389P^ variant generated fewer late-stage neurons, including mature dendrites, suggesting roles in differentiation and survival [5]. Mice harboring the equivalent Crlf3 variant possessed decreased dendrite lengths and branching, along with electrophysiologic defects observed in hippocampal neurons [11], with abnormal behaviors and tremors noted in a *Crlf3* knockout mouse [12]. However, expression of ganglion markers during embryogenesis was not impacted in *crfl3* knockout zebrafish [7], suggesting the neuronal effects might be limited to adults.

CRLF3 has additionally been associated with the neuroprotective effects mediated by EPO. Recombinant human (rh)EPO has been shown to mediate neuroprotection in insects against hypoxia and other insults via the JAK–STAT pathway [21], but had no effect in the fruit fly (*Drosophila melanogaster*), an insect that lacks CRLF3 [13]. This rhEPO-mediated neuronal survival was abolished by RNA interference (RNAi)-mediated *crfl3* ablation in brain-derived neurons from both the red flower beetle (*Tribolium casta*) [13] and migratory locust [3]. The effects of rhEPO could be replaced by locust-derived hemolymph in both beetle and locust cultures, suggesting the existence of an endogenous ligand, with this effect abolished by *crlf3* RNAi [4]. Further studies revealed that EPO/CRLF3 was able to block the hypoxia-induced increase in acetylcholinesterase (ACE) gene expression that mediates apoptosis [22]. The neuroprotective role was also recapitulated in neurons derived from human iPSCs, with *CRLF3* ablation leading to the loss of neuroprotection mediated by a specific EPO isoform generated by alternative splicing [14].

### 2.2. Hematopoietic Cells

Expression of *CRLF3* gene homologues has been consistently observed in several hematopoietic locations. During embryogenesis, zebrafish *crlf3* was shown to be expressed in regions of the lateral plate mesoderm that serve as sites of myelopoiesis during primitive hematopoiesis, as well as the developing thymus, the location of T lymphocyte development during the early definitive phase of hematopoiesis [7]. This was consistent with studies of the mouse *Crlf3* homologue that showed expression in the fetal liver, a transient site of hematopoiesis during embryogenesis [18]. In adults, migratory locust *crlf3* was expressed in innate immune cells called hemocytes [3]; in zebrafish, *crlf3* was expressed across multiple hematopoietic populations in the kidney and spleen [23,24]; in the brown croaker, *crlf3* was expressed in the kidney and spleen [17]; in the African clawed frog, *crlf3* was expressed in the thymus [19]; in the mouse, *Crlf3* was expressed in the thymus and spleen [18]; and in the human, *CRLF3* was expressed in the thymus, spleen and bone marrow [25], which is particularly high in neutrophils and lymphoid cells [20].

Further studies have demonstrated that CRLF3 plays a broad role in hematopoietic cell development and function. Ablation of zebrafish *crlf3* impacted primitive hematopoiesis at the level of hematopoietic progenitors, resulting in a reduction in the primitive myeloid and erythroid lineages, with myeloid cells most impacted. During early definitive hematopoiesis, the number of hematopoietic stem cells (HSCs) was decreased in *crlf3* mutants with a concomitant reduction in the numbers of monocytes, neutrophils and mature T lymphoid cells also observed, although again the effect on myeloid cells was more substantial [7]. Consistent with this, human *CRLF3* gene variants have been associated with an increased percentage of lymphocytes relative to other white blood cells in circulation [6]. Studies in the chicken (*Gallus gallus*) identified *crlf3* as one of a group of five genes strongly associated with adaptive immune responses, with an additional weaker association with innate immune responses [16]. In the teleost species, brown croaker *crlf3* was shown to be a negative regulator of type I interferon (IFN) production in response to viral infection. It was induced by a virus or viral mimics in the major hematopoietic organ, the kidney, with its overexpression able to suppress type I IFN production, while its silencing led to enhanced IFN production and reduced viral replication [17].

Genetic ablation of mouse *Crlf3* did not impact immune cell populations, but resulted in thrombocytopenia that was associated with increased megakaryocytes and production of pre-platelets, but inhibited maturation into mature platelets, although the platelets produced showed normal function [6]. The *Crlf3* knockout mice also showed significant alterations in several red blood cell parameters, including decreased mean corpuscular volume (MCV), mean cell hemoglobin concentration (MCHC), and increased RBC distribution width [12,15].

### 2.3. Other Tissues

*CRLF3* expression has also been identified at various other sites. This includes adaxial cells within the pre-somitic mesoderm and developing pancreas during zebrafish embryogenesis [7]. In adults, expression has additionally been reported in migratory locust skeletal muscle [3] and the heart, liver and intestine of the brown croaker [17], the African clawed frog testes [19], mouse ovaries, testes, liver and other visceral organs [18] and syncytial trophoblasts in humans [20]. *Crlf3* knockout mice displayed reduced overall body weight and, in particular, reduced lean body mass [12]. However, expression of key markers of skeletal muscle development was normal during zebrafish embryogenesis, with no obvious differences in growth observed in *crlf3* mutants [7], so the role of CRLF3 in other tissues remains unclear.

## 3. Functional Context of CRLF3

**Figure 2:**
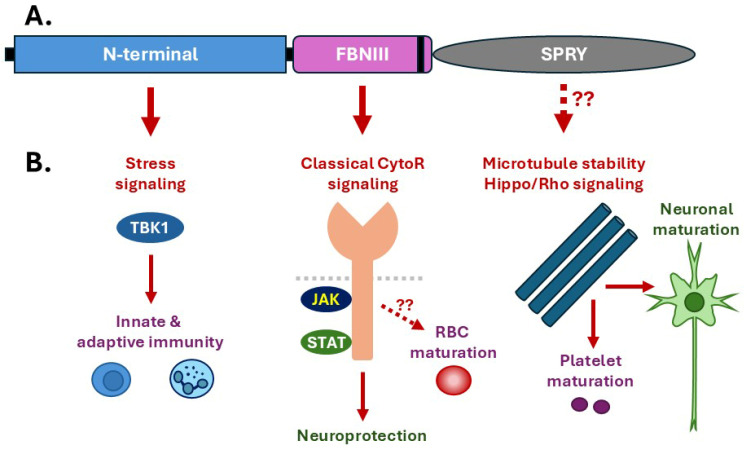
Structure and function of CRLF3. Schematic representation of the constituent structural elements of CRLF3, including a poorly defined N-terminal region, as well as a fibronectin type III (FBNIII) domain containing a WSXWS sequence (thick black line) and a spla and ryanodine receptor (SPRY) domain confirmed by X-ray crystallography (PBD ID 6RPX) (**A**). Potential functional modes by which CRLF3 exerts its various biological roles are also indicated, mapped to the relevant structural element (**B**).

The mechanism of action for CRLF3 remains controversial, with its structural make-up yet to be fully elucidated (Figure 2A), as is its site of action in the cell, with both cytoplasmic and cell membrane locations described [26]. In addition, CRLF3 has been shown to interact with diverse proteins depending on the cellular context. Together, these studies suggest several different mechanisms, which are not necessarily mutually exclusive. Key among these are its possible involvement in classical cytokine signaling, microtubule stability and stress signaling (Figure 2B).

### 3.1. Structural Information

The human *CRLF3* gene is located on chromosome 17q11.2, comprising eight exons spread across 42 kb, with several splice variants and at least three pseudogenes on other chromosomes [27,28]. The major transcript encodes a 442-residue protein, with alternatively spliced forms encoding modified versions of CRLF3, but these have not been studied in any detail. Moreover, the exact domain structure of the various CRLF3 proteins remains contentious. The homology between CRLF3 and class I cytokine receptors has been recognized for some time, with CRLF3 placed in a subgroup that notably includes the EPO receptor [29]. Cytokine receptors are composed of a number of different domains, but all share one or more cytokine receptor homology domains (CHD), along with additional extracellular domains as well as a transmembrane and intracellular region [1]. Each CHD consists of tandem fibronectin type III (FBNIII) domains, structures composed of up to seven β-strands arranged in two anti-parallel β-sheets in a β-sandwich fold, with the second FBNIII domain in class I cytokine receptors containing a WSXWS motif [1,29]. This motif has been implicated in efficient folding and passage through the endoplasmic reticulum to the Golgi [30], but also acts as a conformational switch during receptor activation [31]. It is universally accepted that CRLF3 possesses a central WSXWS-containing FBNIII domain (residues 179–273), which has been confirmed by X-ray crystallography [6]. It has been suggested that CRLF3 may possess a complete CHD [1,29], but other predictions indicate that the N-terminal region is strongly alpha-helical with residues 10–57 projected to form a coiled-coil domain (CCD) with a propensity for membrane interactions, although no signal peptide for mediating translocation to the extracellular surface has been identified [32]. Recent crystallographic studies have suggested that the C-terminus of CRLF3 (residues 274–442) is a spla and ryanodine receptor (SPRY) domain [6]. This domain represents a bent β-sandwich comprising two β-sheets involving ten β-strands found in many eukaryotic proteins, where it acts as a protein–protein interaction module used by proteins involved in RNA processing, histone H3 methylation regulation, innate immunity and embryonic development [33].

### 3.2. Functional Modalities

#### 3.2.1. Classical Cytokine Receptor Signaling

Signaling via cytokine receptors is one of the major pathways for cell-to-cell communication, particularly in the development of blood and immune cells [34,35]. Cytokine receptors are expressed on the surface of responsive cells, with distinct extracellular, transmembrane and intracellular regions. Activation is achieved through binding of their cognate cytokine to the extracellular region, initiating intracellular signal transduction [36], with the Janus kinase–signal transducer and activator of the transcription (JAK–STAT) pathway used as a key mechanism to mediate intracellular signaling and appropriate cellular responses [35].

Several lines of evidence suggest that CRLF3 functions in a classical cytokine signaling mode. Firstly, it possesses a hallmark WSXWS-containing FBNIII domain that shows homology with known cytokine receptors [29], the predicted structure of which has been confirmed experimentally [6]. Secondly, studies in insect systems have demonstrated a CRLF-dependent neuroprotective function mediated by alternatively spliced variants of the mammalian cytokine EPO [4], with recent confirmation of a similar function for human CRLF3 [14]. Thirdly, insect CRLF3 has been shown to lie upstream of the JAK–STAT pathway [37], and STAT3 was identified as a downstream mediator of human CRLF3 in HEK293 cells [28]. Finally, unbiased scRNAseq placed CRLF3 within an expression cluster, ‘lymphoid tissue–cytokine signaling’ [20].

There is also circumstantial evidence that supports this functional mode. Many of the phenotypes resulting from CRLF3 modulation impact blood and immune cell populations, which are those most influenced by cytokine receptor signaling [1]. Moreover, CRLF3 is most closely related to thrombopoietin receptor (TPOR), the main regulator of platelet production [38], and EPOR, the principal mediator of red blood cell (RBC) homeostasis [39] but also linked to the production of larger platelets [40], and interleukin 3 receptor beta common (IL-3Rβc), a key component of receptors that modulate innate immune responses [41], sharing a common ancestor [42]. Furthermore, an activated form of JAK2, which lies downstream of both TPOR and EPOR, could normalize the platelet count in CRLF3-deficient mice [6]. Collectively, this provides plausibility for CRLF3 utilizing a cytokine receptor signaling modality to impact hematopoietic cells. EPO signaling has also been implicated in tissue protection, especially neuroprotection [43], including that caused by hypoxia [44], via impacts on antioxidant signaling [45] and mitochondrial metabolism [46]. This has been shown to be mediated by heteromeric receptors involving EPOR and alternative cytokine receptor chains, including the interleukin-3 beta common chain (IL-3Rβc) [47]. Notably, alternatively spliced forms of EPO have been identified that are specific for tissue protection, consistent with a different binding paradigm [14,21,48]. It is therefore conceivable that CRLF3 represents an alternative to EPOR for mediating neuroprotection in invertebrates and/or a potential alternative heterodimeric partner for EPOR in vertebrates.

#### 3.2.2. Microtubule Stability via Hippo/Rho Signaling

A distinct role for CRLF3 has been identified in the regulation of cell and organelle morphogenesis, with its ablation impacting neuronal outgrowth [5] and synaptic vesicle biogenesis [10], as well as the release of mature platelets from large pre-platelet precursors [6]. The neuronal effects have been associated with disrupted RhoA signaling [5], which has been shown to regulate microtubule stability and dynamics [49]. The platelet effects appear to be a consequence of increased microtubule stability mediated through enhanced polyglutamylation of tubulins [6]. CRLF3 was enriched in platelet sub-cellular fractions containing cytoskeletal proteins, including α-tubulin, although in pre-platelet producing megakaryocytes, it was membrane-associated [6]. These effects are thought to be due to interaction with components of the Hippo pathway, an evolutionarily conserved pathway known to regulate organ size [50]. Amongst the pathway components, CRLF3 interacted with STK38 directly and MOB1 indirectly, with MOB1 phosphorylation associated with enhanced tubulin stability [6]. Interestingly, STK38 and MOB1 variants have been implicated in mean platelet volume (MPV) and mean platelet count (MPC), respectively [6]. CRLF3 was also found to associate with MOB1 in HEK cells treated with okadaic acid [51,52]. However, MOB1 interacts with the Rho guanine exchange factors DOCK6-8 [52], suggesting these pathways may interact. Exactly which region(s) of CRLF3 is responsible for this modality remains unclear, with many of the key studies made in the context of full-length CRLF3 [5,6,10,51]. However, given that the SPRY domain functions as a protein interaction domain, it is likely that it is involved in facilitating this functional mode. Indeed, the CRLF3^L389P^ variant that impacts neuronal development and function lies in the SPRY domain [5]. Moreover, another SPRY-domain containing protein, SPRY and SOCS box 2 (SPSB2), has been separately implicated in platelet production [53].

#### 3.2.3. Stress Signaling

Finally, the role of CRLF3 in innate and adaptive immunity may be part of a stress response. CRLF3-mediated inhibition of viral-induced IFN was shown to be mediated by interaction via its N-terminal domain with Tank binding kinase 1 (TBK1), which is involved in phosphorylation of targets in the autophagy pathway and IFN induction [54]. CRLF3 binding promoted degradation of TBK1 via K48-linked ubiquitination to suppress this effect [17]. It is unclear how this aspect is mediated, although it is worth noting that TRIM family members that contain the SPRY domain in tandem with a RING E3 ligase are implicated in limiting the effects of virus-induced type I IFN [33].

### 3.3. Evolutionary Considerations

The various functions and proposed mechanisms may reflect a sequential functional diversification during evolution. The emergence of CRLF3 coincided with the genesis of the traditional nervous system [3], suggesting an initial function in neuronal development and/or function, presumably mediated by impacts on microtubule assembly. This is supported by the presence of *crlf3* within the genome of a placozoan species (*Trichoplax adhaerens*), which has neurosecretory cells despite lacking a traditional nervous system and a functional Hippo signaling system [55]. It has been suggested that CRLF3 forms part of an ancient cell-protection system that responds to various stresses initially in the nervous system and later as part of innate immunity, including mediating cross-talk between the blood and nervous system [3].

In contrast, the complete cytokine signaling pathway coalesced much later in evolution within the Bilateria, with CRLF3 becoming a core part of the pathway. This underpins the neuroprotective function of CRLF3 and presumably its functional expansion into regulating the development and function of blood and immune cells. Finally, its role in platelet biology is necessarily a recent innovation, since the formation of platelet fragments from a large polyploid megakaryocyte precursor only originated in mammals around 225 MYA [56]. A precedent exists for cytokine receptors to be co-opted into such non-canonical roles, for example, in the extrinsic coagulation pathway, which is thought to have emerged in jawless fish around 450 MYA. In this case, tissue factor (TF), which retains the class II cytokine receptor structure with two FBNIII domains, a transmembrane and cytoplasmic region, but whose ligand is factor VIIa rather than a cytokine that triggers signaling via alternative intracellular pathways [57].

## 4. Involvement of CRLF3 in Human Diseases

### 4.1. Neurological Disorders

The human *CRLF3* gene was first identified (as cytokine receptor-related molecule 4 or *CYTOR4*) in the context of neurofibromatosis type 1 (NF1), a disease characterized by benign nerve tumors called neurofibromas in concert with melanocyte and skeletal defects, as well as cancer susceptibility [58]. In this context, *CRLF3* was discovered to be one of fourteen genes and four microRNAs deleted in a cohort of NF1 patients with more severe disease, including developmental delay, severe autism and cognitive deficits [59]. Recent studies have implicated *CRLF3* in the autistic aspects of this disease, with patients with the deleterious CRLF3^L389P^ variant displaying a higher autistic trait burden [5]. Alternatively, CRLF3 expression in the prefrontal cortex has been found to be a predictor of Alzheimer’s disease [60]. It has also been flagged as a potential contributor to a predisposition to amyotrophic lateral sclerosis (ALS) [61], a degenerative motor neuron disease leading to loss of muscle functionality and other health issues [62]. Single-cell sequencing studies identified expression of CRLF3 and five other genes to negatively correlate with internalizing psychiatric symptoms in a patient cohort at high risk for mental health conditions [63], while GWAS studies have additionally identified CRLF3 as one of four genes associated with epigenetic age acceleration across multiple brain regions [64].

### 4.2. Hematological Disorders

A number of studies have suggested potential roles in immunity/inflammation. Thus, *CRLF3* expression has been identified as a potential biomarker in chronic obstructive pulmonary disease (COPD), a disorder associated with enhanced type 1 and 3 immune responses [65]. It has also been recognized as a risk locus for cutaneous leishmaniasis, being highly expressed in the resultant skin lesions that develop following Leishmania infection [8]. Variants of CRLF3 are also associated with altered platelet parameters in humans [6].

### 4.3. Other Diseases

CRLF3 has been further implicated in various other diseases, notably including neoplasia, especially carcinomas. Typically, the changes relate to increased expression and/or gain in copy number, including in over 60% of ovarian and renal cell carcinomas [66], as well as in a significant proportion of head and neck squamous cell carcinomas and pancreatic ductal acinar carcinomas [67]. In more detailed studies, upregulation of *CRLF3* gene expression was identified in non-melanoma skin cancer and cutaneous squamous cell carcinoma (CSCC), as well as its precursor, actinic keratosis (AK) [68]. Similarly, both *CRLF3* mRNA and the encoded protein were shown to be increased in liver hepatocellular carcinoma (LIHC), with patients possessing elevated *CRLF3* showing reduced overall and disease-specific survival, suggesting it could potentially serve as a prognostic marker [9]. As a corollary, CRLF3 was found to be upregulated in liver cirrhosis mediated by hepatitis C virus (HCV), a leading cause of LIHC [69]. CRLF3 protein and antibodies were collectively increased in breast cancer (principally invasive carcinoma), with CRLF3 antibodies significantly elevated in early-stage breast cancer compared to controls, in HER2+ versus luminal A forms, and in both of these versus triple-negative (TN) forms [70]. Increased *CRLF3* expression has also been observed in leukemia [28] and lymphoma [67], while a rare *UTP6–CRLF3* fusion has additionally been identified in acute myeloid leukemia, although its role in oncogenesis remains unclear [71]. However, the impacts of CRLF3 are not universal, since some cancer types are associated with reduced expression or mutations that are predicted to be loss-of-function [72]. This was supported by early studies in HEK293T cells, demonstrating CRLF3’s function as a negative regulator of proliferation by inhibiting entry into the S phase of the cell cycle [28]. Finally, *SUZ12P1–CRLF3* fusion transcripts have been identified to be enriched in people of African-American or Nigerian descent and associated with acute cardiovascular disease [73].

## 5. Conclusions

Despite its discovery and recognition as a cytokine receptor homologue over 20 years ago, knowledge about CRLF3 has remained remarkably rudimentary. However, a raft of recent studies has greatly enhanced our understanding of CRLF3 structure, function, evolution, potential mechanisms of action and role in disease. These studies have revealed divergent functions, including roles in neuronal development and neuroprotection, as well as innate and adaptive immunity and maturation of platelets and red blood cells. Disruption of CRLF3 has been associated with neurological disorders, while its overexpression has been observed in a range of cancers. The structure–function relationships remain ambiguous, with the different functions of CRLF3 seemingly mediated by alternative modes of action, with recognition that classical cytokine signaling, Hippo/Rho signaling and stress-related signaling might all be important in specific contexts. Key knowledge gaps include confirmation of its role as a classical cytokine receptor, including identification of its native ligand(s) and characterization of ligand–receptor interactions and downstream signaling, as well as verification of a functional SPRY domain and mapping of the proteins with which it may interact. Further studies using the recently described mouse and zebrafish knockout models and patient-derived ex vivo organoids in concert with classical cell biology and biochemical investigations are anticipated to provide further insights and clarity regarding this intriguing protein.

## Figures and Tables

**Table 1:** Phenotypes attributed to CRLF3.

	Phenotype	Species	System	Study	Refs
**Neuronal**	Neuronal maturation defect	Rat	Neuronal cell line	Crlf3 KO (gene trap)	[10]
		Mouse	Whole animal	Crlf3^L389P^	[11]
		Human	iPSC-cerebral organoid	CRLF3^L389P^ + CRLF3 KD	[5]
	Behavioral defects & tremors	Mouse	Whole animal	Crlf3 KO	[12]
	Neuroprotection	Red flour beetle	Brain-derived neurons	crlf3 KD (RNAi)	[13]
		Migratory locust	Brain-derived neurons	crlf3 KD (RNAi)	[4]
		Human	iPSC-derived neurons	CRLF3 KO	[14]
**Hematopoietic/immune**	Platelet maturation defect	Mouse	Whole animal	Crlf3 KO	[6]
	RBC maturation defect	Mouse	Whole animal	Crlf3 KO	[12,15]
	HSC decrease	Zebrafish	Whole animal	crlf3 KO	[7]
	Myeloid cell decrease	Zebrafish	Whole animal	crlf3 KO	[7]
	Lymphoid cell increase	Human	Blood samples	CRLF3 SNV association	[6]
	Adaptive immunity	Chicken	Whole animal	crlf3 SNV association	[16]
	Innate immunity	Chicken	Whole animal	crlf3 SNV association	[16]
	Type I IFN responses	Brown croaker	Kidney cells	crlf3 KD + OE	[17]
	Leishmaniasis risk	Human	Case/control data	CRLF3 SNV association	[8]

Abbreviations: HSC, hematopoietic stem cell; IFN, interferon; iPSC, induced pluripotent stem cell; KD, knock-down; KO, knockout; OE, overexpression; RBC, red blood cell; RNAi, RNA interference; SNV, single nucleotide variant.

## Data Availability

No new data were created or analyzed in this study. Data sharing is not applicable to this article.

## References

[1] Liongue C., Ward A.C. (2007). Evolution of class I cytokine receptors. BMC Evol. Biol..

[2] Wyder S., Kriventseva E.V., Schroder R., Kadowaki T., Zdobnov E.M. (2007). Quantification of ortholog losses in insects and vertebrates. Genome Biol..

[3] Hahn N., Buschgens L., Schwedhelm-Domeyer N., Bank S., Geurten B.R.H., Neugebauer P., Massih B., Gopfert M.C., Heinrich R. (2019). The orphan cytokine receptor CRLF3 emerged with the origin of the nervous system and is a neuroprotective erythropoietin receptor in locusts. Front. Mol. Neurosci..

[4] Knorr D.Y., Hartung D., Schneider K., Hintz L., Pies H.S., Heinrich R. (2021). Locust hemolymph conveys erythropoietin-like cytoprotection via activation of the cytokine receptor CRLF3. Front. Physiol..

[5] Wegscheid M.L., Anastasaki C., Hartigan K.A., Cobb O.M., Papke J.B., Traber J.N., Morris S.M., Gutmann D.H. (2021). Patient-derived iPSC-cerebral organoid modeling of the 17q11.2 microdeletion syndrome establishes CRLF3 as a critical regulator of neurogenesis. Cell Rep..

[6] Bennett C., Lawrence M., Guerrero J.A., Stritt S., Waller A.K., Yan Y., Mifsud R.W., Ballester-Beltran J., Baig A.A., Mueller A. (2022). CRLF3 plays a key role in the final stage of platelet genesis and is a potential therapeutic target for thrombocythaemia. Blood.

[7] Taznin T., Perera K., Gibert Y., Ward A.C., Liongue C. (2022). Cytokine receptor-like factor 3 (CRLF3) contributes to early zebrafish hematopoiesis. Front. Immunol..

[8] Castellucci L.C., Almeida L., Cherlin S., Fakiola M., Francis R.W., Carvalho E.M., Santos da Hora A., do Lago T.S., Figueiredo A.B., Cavalcanti C.M. (2021). A genome-wide association study identifies SERPINB10, CRLF3, STX7, LAMP3, IFNG-AS1, and KRT80 as risk loci contributing to cutaneous Leishmaniasis in Brazil. Clin. Infect. Dis..

[9] Huang Z., Huang L., Huang C., Zhang X., Zhang X. (2025). Expressional and prognostic value of CRLF3 in liver hepatocellular carcinoma patients via integrated bioinformatics analyses and experiments. Res. Sq..

[10] Hashimoto Y., Muramatsu K., Kunii M., Yoshimura S., Yamada M., Sato T., Ishida Y., Harada R., Harada A. (2012). Uncovering genes required for neuronal morphology by morphology-based gene trap screening with a revertible retrovirus vector. FASEB J..

[11] Wilson A.F., Barakat R., Mu R., Karush L.L., Gao Y., Hartigan K.A., Chen J.K., Shu H., Turner T.N., Maloney S.E. (2023). A common single nucleotide variant in the cytokine receptor-like factor-3 (CRLF3) gene causes neuronal deficits in human and mouse cells. Hum. Mol. Genet..

[12] Bult C.J., Blake J.A., Smith C.L., Kadin J.A., Richardson J.E., Mouse Genome Database G. (2019). Mouse Genome Database (MGD) 2019. Nucleic Acids Res..

[13] Hahn N., Knorr D.Y., Liebig J., Wustefeld L., Peters K., Buscher M., Bucher G., Ehrenreich H., Heinrich R. (2017). The insect ortholog of the human orphan cytokine receptor CRLF3 is a neuroprotective erythropoietin receptor. Front. Mol. Neurosci..

[14] Knorr D.Y., Rodriguez Polo I., Pies H.S., Schwedhelm-Domeyer N., Pauls S., Behr R., Heinrich R. (2023). The cytokine receptor CRLF3 is a human neuroprotective EV-3 (Epo) receptor. Front. Mol. Neurosci..

[15] Dickinson M.E., Flenniken A.M., Ji X., Teboul L., Wong M.D., White J.K., Meehan T.F., Weninger W.J., Westerberg H., Adissu H. (2016). High-throughput discovery of novel developmental phenotypes. Nature.

[16] Polewko-Klim A., Lesinski W., Golinska A.K., Mnich K., Siwek M., Rudnicki W.R. (2020). Sensitivity analysis based on the random forest machine learning algorithm identifies candidate genes for regulation of innate and adaptive immune response of chicken. Poult. Sci..

[17] Yan X., Zheng W., Geng S., Zhou M., Xu T. (2023). Cytokine receptor-like factor 3 negatively regulates antiviral immunity by promoting the degradation of TBK1 in teleost fish. J. Virol..

[18] Smith C.M., Hayamizu T.F., Finger J.H., Bello S.M., McCright I.J., Xu J., Baldarelli R.M., Beal J.S., Campbell J., Corbani L.E. (2019). The mouse gene expression database (GXD): 2019 update. Nucleic Acids Res..

[19] Karimi K., Fortriede J.D., Lotay V.S., Burns K.A., Wang D.Z., Fisher M.E., Pells T.J., James-Zorn C., Wang Y., Ponferrada V.G. (2017). Xenbase: A genomic, epigenomic and transcriptomic model organism database. Nucleic Acids Res..

[20] Karlsson M., Zhang C., Mear L., Zhong W., Digre A., Katona B., Sjostedt E., Butler L., Odeberg J., Dusart P. (2021). A single-cell type transcriptomics map of human tissues. Sci. Adv..

[21] Miljus N., Massih B., Weis M.A., Rison J.V., Bonnas C.B., Sillaber I., Ehrenreich H., Geurten B.R., Heinrich R. (2017). Neuroprotection and endocytosis: Erythropoietin receptors in insect nervous systems. J. Neurochem..

[22] Knorr D.Y., Schneider K., Buschgens L., Forster J., Georges N.S., Geurten B.R.H., Heinrich R. (2022). Protection of insect neurons by erythropoietin/CRLF3-mediated regulation of pro-apoptotic acetylcholinesterase. Sci. Rep..

[23] Carmona S.J., Teichmann S.A., Ferreira L., Macaulay I.C., Stubbington M.J., Cvejic A., Gfeller D. (2017). Single-cell transcriptome analysis of fish immune cells provides insight into the evolution of vertebrate immune cell types. Genome Res..

[24] Tang Q., Iyer S., Lobbardi R., Moore J.C., Chen H., Lareau C., Hebert C., Shaw M.L., Neftel C., Suva M.L. (2017). Dissecting hematopoietic and renal cell heterogeneity in adult zebrafish at single-cell resolution using RNA sequencing. J. Exp. Med..

[25] Uhlén M., Fagerberg L., Hallström B.M., Lindskog C., Oksvold P., Mardinoglu A., Sivertsson Å., Kampf C., Sjöstedt E., Asplund A. (2015). Tissue-based map of the human proteome. Science.

[26] Zhao Y., Duan S.S., Che C.Y., Zhang L., Zhang Y., Wang S.H., Liu L. (2008). The expression of human cytokine receptor-like factor 3 in mammalian and *E. coli* cells. Xi Bao Yu Fen. Zi Mian Yi Xue Za Zhi.

[27] Jenne D.E., Tinschert S., Dorschner M.O., Hameister H., Stephens K., Kehrer-Sawatzki H. (2003). Complete physical map and gene content of the human NF1 tumor suppressor region in human and mouse. Genes Chromosomes Cancer.

[28] Yang F., Xu Y.P., Li J., Duan S.S., Fu Y.J., Zhang Y., Zhao Y., Qiao W.T., Chen Q.M., Geng Y.Q. (2009). Cloning and characterization of a novel intracellular protein p48.2 that negatively regulates cell cycle progression. Int. J. Biochem. Cell Biol..

[29] Boulay J.L., O’Shea J.J., Paul W.E. (2003). Molecular phylogeny within type I cytokines and their cognate receptors. Immunity.

[30] Hilton D.J., Watowich S.S., Katz L., Lodish H.F. (1996). Saturation mutagenesis of the WSXWS motif of the erythropoietin receptor. J. Biol. Chem..

[31] Dagil R., Knudsen M.J., Olsen J.G., O’Shea C., Franzmann M., Goffin V., Teilum K., Breinholt J., Kragelund B.B. (2012). The WSXWS motif in cytokine receptors is a molecular switch involved in receptor activation: Insight from structures of the prolactin receptor. Structure.

[32] UniProt Consortium (2025). UniProt: The universal protein knowledgebase in 2025. Nucleic Acids Res..

[33] D’Cruz A.A., Babon J.J., Norton R.S., Nicola N.A., Nicholson S.E. (2013). Structure and function of the SPRY/B30.2 domain proteins involved in innate immunity. Protein Sci..

[34] Robb L. (2007). Cytokine receptors and hematopoietic differentiation. Oncogene.

[35] Liongue C., Sertori R., Ward A.C. (2016). Evolution of cytokine receptor signaling. J. Immunol..

[36] O’Sullivan L.A., Liongue C., Lewis R.S., Stephenson S.E.M., Ward A.C. (2007). Cytokine receptor signaling through the Jak/Stat/Socs pathway in disease. Mol. Immunol..

[37] Miljus N., Heibeck S., Jarrar M., Micke M., Ostrowski D., Ehrenreich H., Heinrich R. (2014). Erythropoietin-mediated protection of insect brain neurons involves JAK and STAT but not PI3K transduction pathways. Neuroscience.

[38] Hitchcock I.S., Hafer M., Sangkhae V., Tucker J.A. (2021). The thrombopoietin receptor: Revisiting the master regulator of platelet production. Platelets.

[39] Tichil I., Mitre I., Zdrenghea M.T., Bojan A.S., Tomuleasa C.I., Cenariu D. (2024). A review of key regulators of steady-state and ineffective erythropoiesis. J. Clin. Med..

[40] Hacein-Bey-Abina S., Estienne M., Bessoles S., Echchakir H., Pederzoli-Ribeil M., Chiron A., Aldaz-Carroll L., Leducq V., Zhang Y., Souyri M. (2020). Erythropoietin is a major regulator of thrombopoiesis in thrombopoietin-dependent and -independent contexts. Exp. Hematol..

[41] Borriello F., Galdiero M.R., Varricchi G., Loffredo S., Spadaro G., Marone G. (2019). Innate immune modulation by GM-CSF and IL-3 in health and disease. Int. J. Mol. Sci..

[42] Boulay J.L., Du Pasquier L., Cooper M.D. (2022). Cytokine receptor diversity in the lamprey predicts the minimal essential cytokine networks of vertebrates. J. Immunol..

[43] Ostrowski D., Heinrich R. (2018). Alternative erythropoietin receptors in the nervous system. J. Clin. Med..

[44] Wakhloo D., Scharkowski F., Curto Y., Javed Butt U., Bansal V., Steixner-Kumar A.A., Wustefeld L., Rajput A., Arinrad S., Zillmann M.R. (2020). Functional hypoxia drives neuroplasticity and neurogenesis via brain erythropoietin. Nat. Commun..

[45] Thompson A.M., Farmer K., Rowe E.M., Hayley S. (2020). Erythropoietin modulates striatal antioxidant signalling to reduce neurodegeneration in a toxicant model of Parkinson’s disease. Mol. Cell Neurosci..

[46] Rey F., Ottolenghi S., Giallongo T., Balsari A., Martinelli C., Rey R., Allevi R., Giulio A.M.D., Zuccotti G.V., Mazzucchelli S. (2021). Mitochondrial metabolism as target of the neuroprotective role of erythropoietin in Parkinson’s disease. Antioxidants.

[47] Brines M., Cerami A. (2005). Emerging biological roles for erythropoietin in the nervous system. Nat. Rev. Neurosci..

[48] Leist M., Ghezzi P., Grasso G., Bianchi R., Villa P., Fratelli M., Savino C., Bianchi M., Nielsen J., Gerwien J. (2004). Derivatives of erythropoietin that are tissue protective but not erythropoietic. Science.

[49] Xu Z., Chen Y., Chen Y. (2019). Spatiotemporal regulation of Rho GTPases in neuronal migration. Cells.

[50] Phillips J.E., Zheng Y., Pan D. (2024). Assembling a Hippo: The evolutionary emergence of an animal developmental signaling pathway. Trends Biochem. Sci..

[51] Couzens A.L., Knight J.D., Kean M.J., Teo G., Weiss A., Dunham W.H., Lin Z.Y., Bagshaw R.D., Sicheri F., Pawson T. (2013). Protein interaction network of the mammalian Hippo pathway reveals mechanisms of kinase-phosphatase interactions. Sci. Signal.

[52] Xiong S., Couzens A.L., Kean M.J., Mao D.Y., Guettler S., Kurinov I., Gingras A.C., Sicheri F. (2017). Regulation of protein interactions by mps one binder (MOB1) phosphorylation. Mol. Cell Proteom..

[53] Masters S.L., Palmer K.R., Stevenson W.S., Metcalf D., Viney E.M., Sprigg N.S., Alexander W.S., Nicola N.A., Nicholson S.E. (2005). Genetic deletion of murine SPRY domain-containing SOCS box protein 2 (SSB-2) results in very mild thrombocytopenia. Mol. Cell Biol..

[54] Pilli M., Arko-Mensah J., Ponpuak M., Roberts E., Master S., Mandell M.A., Dupont N., Ornatowski W., Jiang S., Bradfute S.B. (2012). TBK-1 promotes autophagy-mediated antimicrobial defense by controlling autophagosome maturation. Immunity.

[55] Zhu H., Zhou Z., Wang D., Liu W., Zhu H. (2013). Hippo pathway genes developed varied exon numbers and coevolved functional domains in metazoans for species specific growth control. BMC Evol. Biol..

[56] Martin J.F., D’Avino P.P. (2022). A theory of rapid evolutionary change explaining the de novo appearance of megakaryocytes and platelets in mammals. J. Cell Sci..

[57] Davidson C.J., Tuddenham E.G., McVey J.H. (2003). 450 million years of hemostasis. J. Thromb. Haemost..

[58] Jenne D.E., Tinschert S., Reimann H., Lasinger W., Thiel G., Hameister H., Kehrer-Sawatzki H. (2001). Molecular characterization and gene content of breakpoint boundaries in patients with neurofibromatosis type 1 with 17q11.2 microdeletions. Am. J. Hum. Genet..

[59] Kehrer-Sawatzki H., Cooper D.N. (2021). Classification of NF1 microdeletions and its importance for establishing genotype/phenotype correlations in patients with NF1 microdeletions. Hum. Genet..

[60] Sharma A., Dey P. (2021). A machine learning approach to unmask novel gene signatures and prediction of Alzheimer’s disease within different brain regions. Genomics.

[61] Cirulli E.T., Lasseigne B.N., Petrovski S., Sapp P.C., Dion P.A., Leblond C.S., Couthouis J., Lu Y.F., Wang Q., Krueger B.J. (2015). Exome sequencing in amyotrophic lateral sclerosis identifies risk genes and pathways. Science.

[62] Nijs M., Van Damme P. (2024). The genetics of amyotrophic lateral sclerosis. Curr. Opin. Neurol..

[63] Ota V.K., Oliveira A.M., Bugiga A.V.G., Conceicao H.B., Galante P.A.F., Asprino P.F., Schafer J.L., Hoffmann M.S., Bressan R., Brietzke E. (2025). Impact of life adversity and gene expression on psychiatric symptoms in children and adolescents: Findings from the Brazilian high risk cohort study. Front. Psychiatry.

[64] Lu A.T., Hannon E., Levine M.E., Crimmins E.M., Lunnon K., Mill J., Geschwind D.H., Horvath S. (2017). Genetic architecture of epigenetic and neuronal ageing rates in human brain regions. Nat. Commun..

[65] Maghsoudloo M., Azimzadeh Jamalkandi S., Najafi A., Masoudi-Nejad A. (2020). An efficient hybrid feature selection method to identify potential biomarkers in common chronic lung inflammatory diseases. Genomics.

[66] Cancer Genome Atlas Research N., Weinstein J.N., Collisson E.A., Mills G.B., Shaw K.R., Ozenberger B.A., Ellrott K., Shmulevich I., Sander C., Stuart J.M. (2013). The cancer genome atlas pan-cancer analysis project. Nat. Genet..

[67] Ponten F., Jirstrom K., Uhlen M. (2008). The Human Protein Atlas—A tool for pathology. J. Pathol..

[68] Dang C., Gottschling M., Manning K., O’Currain E., Schneider S., Sterry W., Stockfleth E., Nindl I. (2006). Identification of dysregulated genes in cutaneous squamous cell carcinoma. Oncol. Rep..

[69] Ahmad W., Ijaz B., Hassan S. (2012). Gene expression profiling of HCV genotype 3a initial liver fibrosis and cirrhosis patients using microarray. J. Transl. Med..

[70] Luo R., Chong W., Wei Q., Zhang Z., Wang C., Ye Z., Abu-Khalaf M.M., Silver D.P., Stapp R.T., Jiang W. (2021). Whole-exome sequencing identifies somatic mutations and intratumor heterogeneity in inflammatory breast cancer. NPJ Breast Cancer.

[71] Padella A., Simonetti G., Paciello G., Giotopoulos G., Baldazzi C., Righi S., Ghetti M., Stengel A., Guadagnuolo V., De Tommaso R. (2019). Novel and rare fusion transcripts involving transcription factors and tumor suppressor genes in acute myeloid leukemia. Cancers.

[72] Tate J.G., Bamford S., Jubb H.C., Sondka Z., Beare D.M., Bindal N., Boutselakis H., Cole C.G., Creatore C., Dawson E. (2019). COSMIC: The catalogue of somatic mutations in cancer. Nucleic Acids Res..

[73] Elfman J., Goins L., Heller T., Singh S., Wang Y.H., Li H. (2024). Discovery of a polymorphic gene fusion via bottom-up chimeric RNA prediction. Nucleic Acids Res..

